# Targeting gastrointestinal cancers with chimeric antigen receptor (CAR)-T cell therapy

**DOI:** 10.1080/15384047.2022.2033057

**Published:** 2022-02-06

**Authors:** Ross E Staudt, Robert D Carlson, Adam E Snook

**Affiliations:** a Department of Pharmacology & Experimental Therapeutics, Thomas Jefferson University, Philadelphia, PA, USA; b Department of Microbiology & Immunology, Thomas Jefferson University, Philadelphia, PA, USA; c Sidney Kimmel Cancer Center, Thomas Jefferson University, Philadelphia, PA, USA

**Keywords:** Immunotherapy, chimeric antigen receptor (CAR), CAR-T cell therapy, gastrointestinal cancer, colorectal cancer, gastric cancer, esophageal cancer

## Abstract

The immune system is capable of remarkably potent and specific efficacy against infectious diseases. For decades, investigators sought to leverage those characteristics to create immune-based therapies (immunotherapy) that might be far more effective and less toxic than conventional chemotherapy and radiation therapy for cancer. Those studies revealed many factors and mechanisms underlying the success or failure of cancer immunotherapy, leading to synthetic biology approaches, including CAR-T cell therapy. In this approach, patient T cells are genetically modified to express a chimeric antigen receptor (CAR) that converts T cells of any specificity into tumor-specific T cells that can be expanded to large numbers and readministered to the patient to eliminate cancer cells, including bulky metastatic disease. This approach has been most successful against hematologic cancers, resulting in five FDA approvals to date. Here, we discuss some of the most promising attempts to apply this technology to cancers of the gastrointestinal tract.

## Early history of cancer immunology

A nearly two-century long period of “experimental immunology” ushered in much of the foundations related to the understanding of the immune system. Although scholars have identified records of patients being inoculated with smallpox as far back as early Chinese antiquity, the English physician, Edward Jenner, is largely credited as the initial pioneer of vaccination in the late 1700s.^1^ Jenner’s work documented the first scientific attempts to inoculate an individual with an infectious agent to control a corresponding infectious disease. Although Jenner did not fully understand the mechanisms of vaccination and its shaping of immune responses, his efforts would eventually lead to the eradication of smallpox in the 1980s, among several other infectious disease advancements.^2^

Further strides were taken across the subsequent century with the development of what was to become the modern “Germ Theory of Disease” by the separate efforts of Louis Pasteur and Robert Koch. Pasteur, a French chemist originally interested in alcoholic fermentation, correctly identified the source of fermentation, and by extension “spoilage”, as a biological process that manifests from organisms in the air. Koch, a German physician, also observed similar organisms in the blood of sheep afflicted with anthrax. Koch correctly identified that anthrax transmission in animals could occur through exposure and proximity, even from bacterial spores dormant for many years. These findings were the foundation for “Koch’s postulates”, describing the relationship between microbes and disease.^3^ The correct identification of distinct pathogens as the causative agents in infectious disease further spurred a period of rapid vaccine development. Building upon Jenner’s observations of inoculation and conferred immunity, this led some to believe that perhaps cancer too could be stymied through immune modulation.^2^

While Jenner, Pasteur, and Koch established the early dogma of bacteriology and vaccinology, the study of immune modulation of cancer began with independent observations made by two German physicians in the mid-to-late 19^th^ century. Drs. Busch and Fehleisen each observed tumor regression in patients intentionally infected with pathogens responsible for erysipelas.^4^ Shortly thereafter in 1891, an American surgeon, William Coley, developed his cocktail of heat-killed bacteria that was used to treat sarcoma patients with remarkable success, including numerous documented cases of tumor regression following treatment.^5^ Although controversial during that period, Coley’s observations have been validated by our modern understanding of cancer immunology and retrospective analyses.^6,7^

## Immunosurveillance

While the “experimental immunology” era of the 19^th^ century focused primarily on infectious disease and the roles of innate immunity, the 20^th^ century revealed much of what we now know about immunity and cancer. Only sixteen years following Coley’s pivotal observations, Paul Ehrlich formulated a hypothesis that the high frequency of aberrant cell growth and transformation during human development is likely kept in check by “[an] organism’s positive mechanisms.”^8^ Although unable to evaluate this experimentally, Ehrlich’s “positive mechanisms” roughly equated to the presence of an immunological surveillance mechanism actively engaging and eliminating neoplastic cells.^9^

Roughly fifty years later, Ehrlich’s proposition was independently revisited by both Australian, F. MacFarlane Burnet, and American physician, Lewis Thomas. Burnet believed heritable and acquired mutations in somatic cells undergoing abundant proliferation would push cells toward malignancy.^10^ However, these malignant cells would simultaneously possess acquired, and highly specific neoantigens, evoking an immune response that could eliminate those malignant cells.^11^ Thomas supported a similar theory: that complex organisms evolved mechanisms to protect against malignancies using similar mechanisms that resulted in homograft rejection of transplanted tissues.^12^

In hindsight, Ludwik Gross had already evaluated this phenomenon experimentally just over a decade prior. Gross found that low doses of chemically-induced sarcomas could be resected and then transplanted into syngeneic mice leading to periods of tumor growth followed by gradual regression, suggesting an immune response to the tumor. Moreover, rechallenge with high doses of those same sarcomas resulted in outright rejection of tumors due to acquired immunity.^13^ A decade later, E. J. Foley, confirmed Gross’ observations by demonstrating that chemically-induced tumors could be transplanted from one inbred mouse to another, and then subsequently removed, preventing further challenge with transplanted fragments of that same tumor.^14^

Further experimental evidence for Ehrlich’s immunosurveillance hypothesis was reported by Prehn and Main in the 1950s. In their studies, sarcomas induced with the chemical carcinogen MCA were transplanted into partnered syngeneic, naïve mice. Further inoculation of these same mice with sarcomas from the original donors resulted in rejection, however, rechallenge with sarcomas from non-partnered mice resulted in engraftment. Moreover, transplantation of non-transformed skin tissues from the same sarcoma donor mice beforehand did not sensitize the recipient mice to sarcoma engraftment.^15,16^ Prehn and Main’s series of experiments provided evidence that tumors indeed carried a unique antigen “signature”, resulting in tumor rejection by tumor-specific immunity.^5^

## Chimeric antigen receptor (CAR)-T cell therapy

These and other early “immunosurveillance” studies provided the foundation of numerous investigators to search for immunosurveillance in humans,^17^ explore mechanisms of antitumor immunity,^18,19^ and ultimately create immunotherapies to treat cancer.^20^ Those studies revealed T cells as primary mediators of cancer immunity and adoptive transfer of tumor-specific T cells isolated from tumors (tumor-infiltrating lymphocytes; TILs) as a potential therapeutic approach.^20,21^ However, several factors limit the use of TILs as immunotherapeutics, leading to synthetic biology approaches that employ genetically modified peripheral blood lymphocytes with antitumor specificity to potentially mimic TILs.^22^ That approach has evolved into the field of CAR-T cell therapy.

**Figure 1.**
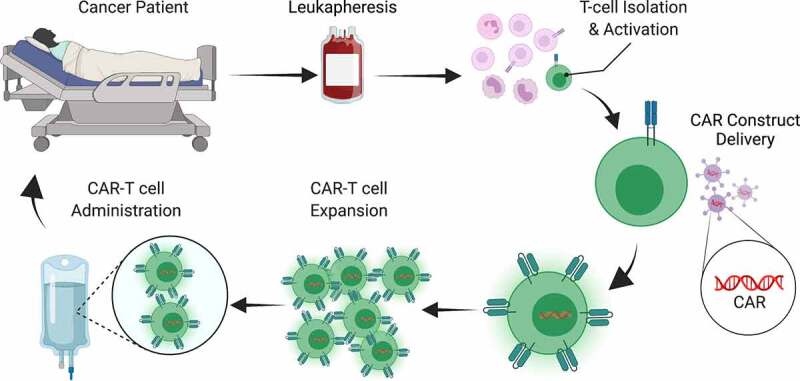
**CAR-T cell manufacturing**. The production of CAR-T cells begins with leukapheresis to collect patient blood cells followed by isolation of T cells. The T cells are then activated and genetically modified to express the CAR, typically by lentiviral transduction. The CAR-T cells are then expanded to large numbers and re-introduced to the patient.

**Figure 2.**
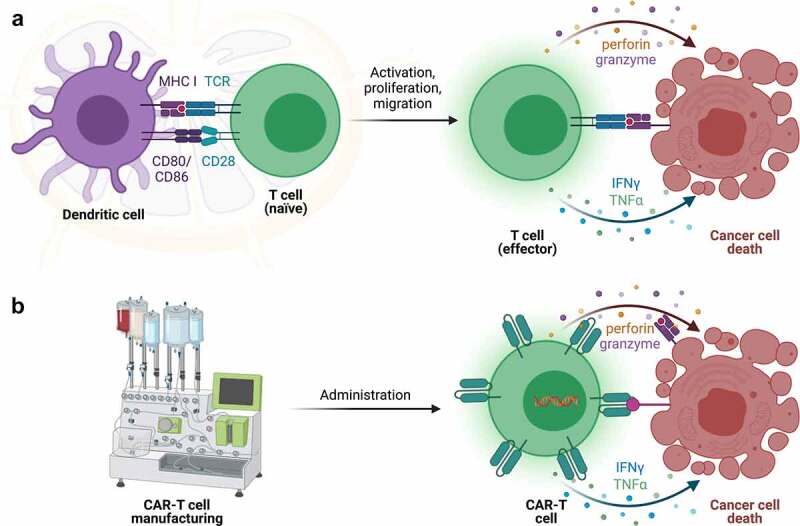
**CAR-T cells overcome some limitations of T cell immunobiology. a**) Naïve T cells require encounter with antigen presenting cells (APCs) possessing antigens on MHC molecules with appropriate costimulatory signals (such as CD80/CD86) in lymph nodes. This induces T-cell differentiation and acquisition of effector functions, such as secretion of cytolytic granules containing perforin and granzyme and production of cytokines (IFNγ, TNFα, and others). Upon encountering the same antigen in the correct MHC molecules on cancer cells, T cells employ those effector mechanisms to induce cancer cell death. **b**) In contrast, CAR-T cells are manufactured in the laboratory and can detect cancer cell targets directly, without the need for MHC molecules, to induce cancer cell death.

A rapidly growing field in cancer immunotherapy are CAR-T cells, which are genetically modified T cells that express a synthetic T-cell receptor to recognize a tumor associated antigen (TAA), leading to cytotoxic T-cell function and subsequent target cell death upon antigen recognition.^23^ The production of CAR-T cells has been well documented but can be briefly summarized here. T cells are collected from a patient’s blood via leukapheresis, genetically modified to express the CAR construct, expanded to large numbers *ex vivo*, and administered back to the same patient (Figure 1).^24^ To better understand CAR-T cells it is important to recognize some biological principles of T cells (Figure 2). Naturally-occurring T cells require 1) activation of their T-cell receptor (TCR) by complexes of target antigen and MHC and 2) engagement of costimulatory signals. Both are provided by specialized immune cells known as antigen presenting cell (APC). Those two interactions induce downstream signaling events that lead to T-cell differentiation that includes acquisition of cytotoxic and inflammatory cytokine effector functions. Upon encountering cancer cells with the same antigen-MHC complexes on their surface, T cells use their effector functions to induce cancer cell apoptosis (Figure 2a).

Importantly, CAR-T cells do not require APCs for their activation (Figure 2b). Instead, this occurs in the laboratory during the manufacturing process. Moreover, the CAR incorporates an antibody-derived, antigen-recognition domain that is attached to a transmembrane domain and intracellular signaling domain.^25,26^ The antibody-derived structure allows the CAR to recognize surface tumor antigens in their native form on tumor cells, without MHC molecules, stimulating production and release of cytotoxic granules and cytokines leading to target cell death.^27,28^ This is important because tumors can avoid immune surveillance by downregulating MHC molecules, which reduces antigen presentation and recognition of tumors cells.^29^ Bypassing antigen presentation eliminates an immune-escape tool from the tumorigenesis tool box and enables an important treatment option when a TAA is present.

First-generation CAR designs employed only CD3ζ chain without additional costimulatory molecules. This design resulted in poor CAR-T cell longevity and efficacy leading to the inclusion of costimulatory domains in future CAR constructs.^29^ Initial 2^nd^-generation designs linked a costimulatory domain of either CD28 (28z) or 4–1BB (BBz) to CD3ζ in the CAR construct.^30^ These designs have proven to be successful in treating hematological malignancies in patients and remain the only the FDA-approved CAR designs to date. The 3^rd^-generation CARs fuse both CD28 and 4–1BB to CD3ζ (28BBz) and are hypothesized to produce long-lived and highly functional CAR-T cells, though a clear clinical benefit of this design over 2^nd^-generation designs has not yet been identified.^30^ The field of CAR design has expanded rapidly in the last five years, beyond the simple 1^st^, 2^nd^, 3^rd^ generation paradigm. CAR-T cell designs may include constitutive or inducible cytokine production,^31^ cytokine signaling domains,^32^ and others. These are intended to improve T-cell activation, proliferation, effector function, longevity, resistance to the hostile tumor microenvironment, and more.

## CAR-T cell therapy for GI cancers

Currently, CAR-T cell therapy is approved for treating certain hematological malignancies, but not any solid tumors. Because CAR-T cell therapy involves administration of very large numbers of highly activated T cells, one of the biggest barriers to this therapy is successfully targeting an antigen to produce robust antitumor immunity without collateral on-target or off-target toxicity in healthy tissues. On-target toxicity to healthy B cells and the resulting B-cell aplasia that arises from CD19-directed CAR T-cell treatment of hematological cancers can be managed clinically, while toxicity to organ systems from other CAR-T cell therapies can be fatal. While no CAR-T cell therapies are FDA-approved to treat solid tumors, there are several clinical trials ongoing and a variety of targets being investigated to treat different gastrointestinal (GI) malignancies.

## Epithelial cell adhesion molecule (EpCAM)

EpCAM is a transmembrane glycoprotein that is involved in proliferation and metastasis.^33^ While EpCAM is important for tumor cell survival it has been shown to reduce cell-to-cell adhesion by reducing E-cadherin, which increases cell motility leading to metastatic disease.^34^ Originally identified in colon cancer,^35^ overexpression of EpCAM has been observed in several different cancers and is, therefore, a potential target for CAR-T cell therapy.^36^ In preclinical trials a third generation EpCAM-targeting CAR-T cell was able to recognize and lyse target cancer cells *in vivo*, delay tumor formation and growth, and avoid inducing on-target, off-tumor toxicity.^37^ This CAR-T cell has since moved to Phase I clinical trials in patients with advanced gastric cancer with peritoneal metastasis (NCT03563326).^38^

## Human epidermal growth factor receptor-2 (HER2)

HER2 is a membrane tyrosine kinase that plays an important role in breast cancer progression and pathogenesis. HER2 is overexpressed in breast cancer cell, is an important prognostic indicator in breast cancer, and has been a major therapeutic target for several decades.^39^ While HER2 has traditionally been linked to breast cancer it is being investigated in various tumor types including GI cancers.^40^ For example, Bellicum Pharmaceuticals is currently investigating the efficacy of its dual-switch HER2-specific CAR-T cell to treat breast cancer and gastric cancer (NCT04650451).^41^ A unique aspect of this CAR-T cell therapy is that the activity of the T cells can essentially be eliminated using a “suicide switch” built into the CAR-T cells to treat or prevent toxicity.^42^ This is potentially critical reflecting the rapid toxicity and death of the first patient to receive a HER2-specific CAR-T cell therapy.^43^ A HER2-specific CAR-T cell clinical trial at Baylor Medical College is monitoring the efficacy of a CAR-T cell regimen to treat several different tumor types including gastric, colorectal, and esophageal cancer (NCT03740256).^44^ This CAR-T cell therapy is unique in that it incorporates an intratumoral oncolytic viral administration that enhances its efficacy in preclinical studies.^45^

## Carcinoembryonic antigen (CEA)

CEA is a cell adhesion glycoprotein that is predominately expressed during fetal development.^46^ CEA is expressed in adult gastrointestinal tissues, predominantly at the luminal surface.^47^ Moreover, CEA is a common TAA that is overexpressed in most colorectal tumors^48^ and detection in serum is a useful biomarker for monitoring colorectal cancer progression.^46^ While the role of CEA in tumor development or progression isn’t clear, CEA can be a therapeutic target. A completed Phase 1 trial of CEA CAR-T cells showed some efficacy in many of the treated patients, while even the highest dose was well-tolerated by patients in this trial.^49^ That research group is currently recruiting for a Phase 2 clinical trial (NCT04348643).^50^

## B7-H3 (CD276)

B7-H3 (CD276) is a transmembrane protein and a member of the B7 family. This family of proteins is necessary for T-cell costimulation, while B7-H3 plays a predominantly inhibitory role in adaptive immunity, suppressing T-cell activation and proliferation.^51^ More importantly, this transmembrane protein is overexpressed in a variety of different cancer types and CAR-T cells targeting B7-H3 have shown positive results in treating pancreatic, ovarian, and brain cancers.^52,53^ Moreover, B7-H3 is overexpressed in esophageal cancer and CAR T-cell therapy effectively targets and treats esophageal squamous cell carcinoma (ESCC) xenografts in mice.^54^ Several B7-H3-directed CAR-T cell therapies are in early-phase clinical trials across a spectrum of adult and pediatric malignancies.

## Claudin18.2

Claudin18.2, a splice variant of claudin 18, is part of a family of proteins that modulate the movement of molecules from cell to cell by interacting with tight junctions. While claudins are present in gastric, pancreatic, and lung tissue, claudin18.2 is specifically expressed in the stomach and, more importantly, it is highly expressed in gastric and gastroesophageal junction (GEJ) adenocarcinoma.^55^ With all cancer targets, the goal is to target cancer cells while ignoring the same target on healthy cells. Claudin18.2 has proven to be a promising target because it is not only highly expressed in carcinomas, but also isolated from therapeutics in healthy tissue because it is embedded in gastric mucosa.^56^

While monoclonal antibodies have been the main approaches to treating claudin18.2+ tumors in the clinic, CAR-T cell therapy clinical trials have recently commenced. Preclinical data provided by CARsgen Therapeutics demonstrated that their claudin18.2-directed CAR-T cell therapy effectively targeted claudin18.2+ patient-derived xenograft (PDX) models of gastric cancer without toxicity.^57^ This therapeutic has since moved to phase 1 clinical trials where it is being tested on patients with gastric and pancreatic cancer in both China (NCT04581473)^58^ and the US (NCT04404595).^59^

## Emerging CAR-T cell therapies for GI cancers

While there are a variety of promising therapeutics currently in clinical trials, they are only the beginning for GI cancer therapies. Guanylyl Cyclase C (GUCY2C) is a transmembrane protein expressed on the luminal surface of intestinal epithelium.^60^ It has become an important target in colorectal cancer^61–63^ and recent data suggests that it can be targeted throughout the GI tract using a variety of approaches,^64,65^ including GUCY2C-directed vaccines.^66,67^ GUCY2C-directed CAR-T cells show efficacy^68,69^ and safety^68^ in animal studies of metastatic colorectal cancer, and PDX model data suggests potential efficacy against gastric and esophageal cancers.^70,71^ GUCY2C-directed CAR-T cell therapies are expected to enter clinical trials in 2022.

MUC1 is another potential CAR-T cell target that has shown promise in targeting GI cancers. MUC1 is an adhesion ligand for stromal and endothelial cells and plays an important role in cancer metastasis.^72^ It is overexpressed and accessible to therapeutics in most epithelial cancers.^73^ There have been attempts to target MUC1+ tumors with CAR-T cells but there has not yet been any success in treating GI cancers. In addition to the above targets, there are many other potential targets for colorectal, gastric, and esophageal cancers in development, making it impossible to discuss them all here.

## Conclusions

The study of immunology has been an evolving field for centuries. Primitive as it was, the initial principles of immunology were created centuries ago and led to the development of vaccines and eradication of life-threatening illnesses. Furthermore, it was these initial principles that not only led to prevention and treatment of bacterial and viral infections, but also discoveries in cancer treatment. It was discovered decades ago that tumor cells possess tumor-specific signatures that can be recognized by the host immune system leading to tumor rejection. Those and other observations laid the foundation for modern cancer immunotherapy.

T cells are critical mediators of natural antitumor immunity which can be leveraged by TIL therapy or immune checkpoint blocking (ICB) therapy, such as antibodies directed against PD-1/L1 and CTLA-4 that established cancer immunotherapy as a pillar of cancer care a decade ago. CAR-T cell therapies go a step farther using a synthetic biology approach to create tumor-directed T cells, rather than rely on endogenous immunity. While there is enormous enthusiasm for this technology, significant work is required to create and identify therapies with sufficient efficacy and acceptable toxicity (clinical, financial, etc). Perhaps one or more of the strategies discussed here will become the first to meet those goals and become the first FDA-approved CAR-T cell therapy for GI cancers.
